# Antioxidant, Antiinflammatory and Antiapoptotic Effects of Lycopene in Rats With Vancomycin‐Induced Nephrotoxicity

**DOI:** 10.1111/bcpt.70084

**Published:** 2025-07-21

**Authors:** Hülya Demirkapı Atik, Orkun Atik, Recep Aslan, Yavuz Osman Birdane, Abdullah Eryavuz

**Affiliations:** ^1^ Department of Physiology, Faculty of Veterinary Medicine University of Afyon Kocatepe Afyonkarahisar Türkiye; ^2^ Department of Pharmacology and Toxicology, Faculty of Veterinary Medicine University of Afyon Kocatepe Afyonkarahisar Türkiye

**Keywords:** apoptosis, inflammation, lycopene, nephrotoxicity, oxidative stress, vancomycin

## Abstract

**Background:**

The most important side effect of vancomycin (Vanco) is nephrotoxicity (NPT). Lycopene (Lyco) has been reported to have anti‐inflammatory and anti‐apoptotic properties in addition to its antioxidant activity. The aim is to investigate the protective efficacy of Lyco against the NPT condition that limits the use of Vanco.

**Method:**

A total of 48 rats were used in the study in six groups of eight rats each, namely, Corn Oil Control, Lyco 5, Lyco 10, Vanco, Vanco + Lyco 5 and Vanco + Lyco 10.

**Results:**

Vanco (400 mg/kg, intraperitoneal) administered for 7 days elevated serum BUN, creatinine, uric acid levels and renal lipid peroxidation while decreasing renal GSH and the activity of the antioxidant enzymes SOD, CAT and GPx. Vanco also increased the levels of inflammatory markers NF‐κB, TNF‐*α*, Bcl‐3 and p38*α* MAPK activity. It decreased the level of AQP‐1 and increased the level of NGAL. In addition, it activated apoptosis by decreasing Bcl‐2 and Procas‐3 expression levels while increasing apoptotic p53, Bax and Cyt‐c expression levels. Lyco treatment at both doses (5 and 10 mg/kg, orally) ameliorated NPT by reducing oxidative stress, inflammation and apoptosis, while the higher dose was more effective.

**Conclusion:**

The findings showed that Lyco attenuated Vanco‐induced NPT.

## Summary

Vancomycin (Vanco) is a bactericidal and tricyclic antibiotic in the class of glycopeptides that acts by suppressing the cell wall synthesis of gram‐positive bacteria. The most important side effect of Vanco is nephrotoxicity (NPT). Because of their protective properties against free oxygen radicals, phytochemicals—in particular, plant phenolics and carotenoids—have garnered more attention recently. Lycopene (Lyco), a non‐cyclic and lipophilic carotenoid, has attracted sizeable scientific interest in recent years. We administered Vanco and Lyco to rats and compared the results. Vanco administration caused damage in rat kidneys, whereas Lyco administration showed antioxidant, antiinflammatory and antiapoptotic activity.

## Graphical abstract

Vancomycin caused nephrotoxicity in rats. Lycopene treatment showed ameliorative efficacy on vancomycin‐induced nephrotoxicity. This efficacy of lycopene was dose dependent.

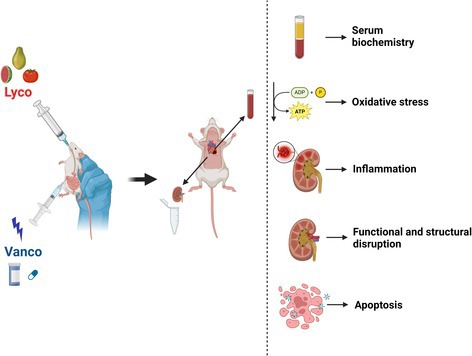

**Abbreviations**
ADS: antioxidant defense system
AQP‐1: aquaporin 1
ATP: adenosine triphosphate
Bax: Bcl‐2‐associated X protein
Bcl‐2: B‐cell lymphoma 2
Bcl‐3: B‐cell lymphoma 3
BUN: blood urea nitrogen
CAT: catalase
CO: corn oil
CRE: creatinine
Cyt‐c: cytochrome c
DNA: deoxyribonucleic acid
ECL: enhanced chemiluminescence
ELISA: enzyme‐linked immunosorbent assay
GPx: glutathione peroxidase
GSH: reduced glutathione
IP: intraperitoneal
IκB: inhibitor kappa B
Lyco: lycopene
MDA: malondialdehyde
MRSA: methicillin‐resistant *staphylococcus aureus*

NF‐κB: nuclear factor kappa B
NGAL: neutrophil gelatinase‐associated lipocalin
NOX‐4: NADPH oxidase 4
NPT: nephrotoxicity
PBS: phosphate‐buffered saline
Procas‐3: procaspase‐3
PVDF: polyvinylidene difluoride
p38*α* MAPK: mitogen‐activated protein kinase p38 alpha
p53: tumor protein 53
ROS: reactive oxygen species
SD: standard deviation
SDS: sodium dodecyl sulfate
SOD: superoxide dismutase
SPSS: statistical package for the social sciences
TBS‐T: tris‐buffered saline with tween
TNF‐*α*
: tumor necrosis factor‐alpha
UA: uric acid
Vanco: vancomycin

## 1 Introduction and Background

Vancomycin (Vanco) is a bactericidal and tricyclic antibiotic in the class of glycopeptides that acts by suppressing the cell wall synthesis of gram‐positive bacteria [1]. Today, despite the availability of alternative drugs, vancomycin is among the preferred antibiotics for methicillin‐resistant *Staphylococcus aureus* (MRSA) infections such as septicemia, endocarditis, meningitis, pneumonia and osteomyelitis. It is also used against bacteria in its spectrum in individuals who are resistant to other antimicrobial drugs and/or allergic to penicillin‐cephalosporins. It leaves the body mainly through glomerular filtration and partially through the kidneys via active tubular secretion [2].

The most important side effect of Vanco that limits its use is nephrotoxicity (NPT). NPT due to Vanco use has been reported to happen in 5%–25% of patients [3]. However, the basis of this undesirable effect is not fully known. As a result of the research, some mechanisms have been revealed, and some of the underlying causes of vancomycin‐induced NPT have been clarified. One of these is that Vanco increases ATP concentrations in renal cells and accordingly triggers oxidative phosphorylation by stimulating oxygen consumption [4]. It is also known that the increase in oxygen consumption leads to free radical production [5]. In addition, in the light of animal experiments, it is stated that proinflammatory oxidation, mitochondrial dysfunction and cellular apoptosis events lie at the basis of NPTs that develop due to Vanco use [6]. Additionally, it has been demonstrated that Vanco directly impacts the renal proximal tubular epithelial cells, causing acute tubulointerstitial injury and ischemia [7].

Recently, interest in phytochemicals, particularly plant phenolics and carotenoids, has increased due to their defensive effects against free oxygen radicals [8]. Carotenoids are made by microbes and plants, and they can take on colours ranging from yellow to red [9]. There are around 700 known carotenoids, of which the human diet contains about 40 [10]. Lycopene, a non‐cyclic and lipophilic carotenoid, has attracted sizeable scientific interest in recent years [11]. The main dietary sources of lycopene for humans are red fruits and vegetables, yet not all red plants are lycopene‐rich.

Tomatoes and tomato‐based products, watermelon, pink grapefruit, papaya, and apricots are the main sources of lycopene (Lyco). Lyco has been rated as “Generally Safe” in the dietary supplement class by the FDA [9]. Lyco is reported to be the most capable singlet oxygen scavenger among all carotenoids [12] and has antioxidant [13], anti‐inflammatory [14] and anti‐apoptotic [15] properties.

The protective and curative efficacy of Lyco against kidney damage induced by various drugs, toxins or xenobiotics has been accepted by researchers [16, 17]. However, no comprehensive study on the protective efficacy of Lyco against NPT caused by Vanco was found in the letters review. The aim of this study was to investigate the protective and curative efficacy of different doses of lycopene treatment against the NPT condition that limits the use of vancomycin, which is used in various disorders.

## 2 Materials and Methods

The study was conducted in accordance with the *Basic & Clinical Pharmacology & Toxicology* policy for experimental and clinical studies [18].

The study was performed on a total of 48 female rats with a body weight of 250–300 g. Rats were kept and fed in an environment with an ambient temperature of 21°C ± 2°C, a humidity level of 55%–60%, and a light–dark cycle of 12 h. Rats were allowed to consume standard pellet feed and water ad libitum, and no special feed or additives were given.

Vanco (Vancomax, VEM Drug) was obtained as a vial containing 1000‐mg lyophilized powder. It was dissolved in saline and administered intraperitoneally. Lyco was obtained as lycopene 10 %FS (DSM Nutritional Products). It was suspended in corn oil (CO) and implemented orally by gastric gavage to the animals with appropriate dosage.

The study was performed in six groups of eight animals each, and the study period was 9 days. In the 1st group (CO Control) rats, corn oil was administered orally (volume: 1 mL) for 8 days. In Groups 2 (Lyco 5) and 3 (Lyco 10), lycopene was suspended in CO at doses of 5 and 10 mg/kg, respectively, and administered orally for 8 days (equal volume). In the 4th group (Vanco), vancomycin was administered intraperitoneally (IP) at a dose of 400 mg/kg once daily for 7 days. In Groups 5 (Vanco + Lyco 5) and 6 (Vanco + Lyco 10), lycopene was applied orally at doses of 5 and 10 mg/kg, respectively, and vancomycin was administered at a dose of 400 mg/kg by IP route 1 day later for 7 days (Lyco administration started 1 day before Vanco administration and was terminated on the same day at the end of the study. Lyco was administered to the rats first, and Vanco was administered 30 min later) [17, 19, 20]. All administrations were performed in the morning hours. 1 day after the last administration, on the ninth day, the rats were sacrificed under isoflurane anaesthesia. After blood was taken from the heart while it was working, the kidneys were also taken using appropriate methods. Blood samples were collected in normal tubes (red capped) for serum, centrifuged at 3000 *g* for 10 min (Heraeus Megafuge 8 R, ThermoFisher Scientific, MA, USA), and the serum portions were separated and stored at −80°C until analysis.

### 2.1 Determination of Kidney Function Parameters

An autoanalyser was used to measure the levels of uric acid (UA), creatinine (CRE) and blood urea nitrogen (BUN) in serum. Serum grades of these markers were decided on an Abbott Architect c8000 machine using Abbott kits.

### 2.2 Determination of Lipid Peroxidation and Antioxidant Defense

Part of the kidney tissues were homogenized in 1.15% potassium chloride solution (pH: 7.4) (1:9 [w/v]) (VDI 12 homogenizer; VWR, PA, USA). After homogenization, the supernatants were centrifuged at 2100 *g* for 10 min at +4°C. The supernatants obtained were stored at −80 °C until the time of analysis. Malondialdehyde (MDA) [21] and reduced glutathione (GSH) [22] levels, superoxide dismutase (SOD) [23] and catalase (CAT) [24] enzyme activities and renal protein amounts [25] were measured by spectrophotometric methods as described previously. Glutathione peroxidase (GPx; item no: E1172Ra, Bioassay Technology Laboratory, Zhejiang, China) and NADPH oxidase 4 (NOX‐4; item no: SEB924Ra, Cloud‐Clone Corp. CCC, TX, USA) enzyme activities were determined by commercial ELISA kits consistent with the manufacturer's instructions. Spectrophotometric measurements were executed using a Shimadzu 1601 UV–VIS spectrophotometer (Tokyo, Japan). ELISA kit assays were measured with the Multiskan FC Microplate Photometer (Thermo, St. Louis, MO, USA).

### 2.3 Determination of Inflammatory Parameters

For inflammatory parameters, kidney tissues were homogenized in PBS buffer (1:9 [w/v]). The samples were centrifuged (Heraeus Megafuge 8 R, ThermoFisher Scientific, MA, USA) at 14000 *g* for 15 min at +4°C. The supernatants obtained were stored at −80°C until the time of analysis. Nuclear Factor kappa B (NF‐κB; product no: SEB824Ra), tumour necrosis factor‐alpha (TNF‐*α*; product no: SEA133Ra) and B‐cell lymphoma 3 (Bcl‐3; Product No: SEB734Ra) levels (Cloud‐Clone Corp. [CCC, TX, USA]) and mitogen‐activated protein kinase p38 alpha ([p38*α* MAPK; Catalogue No: CK‐bio‐25 173], Shanghai Coon Koon Biotech Co Ltd. [Shanghai, China]) activity were decided with commercial ELISA kits consistent with the manufacturer's instructions.

### 2.4 Determination of p53, AQP‐1 and NGAL Levels

Tumour protein 53 (p53; Product No: SEA928Ra), aquaporin 1 (AQP‐1; Product No: SEA579Ra) and neutrophil gelatinase‐associated lipocalin (NGAL; Product No: SEB388Ra) levels were determined with commercial ELISA kits consistent with the manufacturer's (Cloud‐Clone Corp. [CCC, TX, USA]) instructions.

### 2.5 Western Blot Analysis

The remaining parts of the kidney tissues from the treated rats were also used for the analysis of apoptotic proteins. Kidney tissue pieces used for Western blot analysis were decontaminated with PBS immediately after removal. Before being analysed, the decontaminated tissues were quickly frozen in liquid nitrogen and kept at −80°C. At the time of analysis, kidney tissues were thawed at +4°C in xTractor buffer (1:20 [w/v], [Takara Bio, CA, USA]) with ProteoGuard EDTA‐Free Protease Inhibitor Cocktail (1%) (Takara Bio, CA, USA) for protein stabilization; RNase/DNase‐free 2 mL tubes (ThermoFisher Scientific, MA, USA) containing 2.4‐mm metal beads were homogenized in a Mini Bead Mill Homogenizer (VWR, PA, USA). The homogenates were centrifuged at 12000 *g* for 20 min at +4°C. Protein concentrations in the supernatants obtained after centrifugation were determined using the BCA Protein Assay Kit (Takara Bio, CA, USA). Samples were diluted with PBS and 4xLaemmli sample buffer (Bio‐Rad, CA, USA) to contain 40‐μg protein per well. To ensure the denaturation of proteins, each sample was incubated in a dry block heater (HB120‐S, DLAB, Johor, Malaysia) with 1‐μL 2‐mercaptoethanol (Bio‐Rad, CA, USA) for 5 min at 95°C. Samples were separated by gel electrophoresis in 10% Tris/Glycine/SDS buffer. Proteins were detached according to molecular size and transferred to PVDF membranes using transfer buffer (Tris/Glycine [10%] + methanol [20%] + distilled water [70%]). After the proteins were transferred to the membranes, they were blocked with blocking buffer ([fish gelatin], [Takara Bio, CA, USA]) for 90 min. At the end of blocking, the membranes were washed three times for 5 min each with TBS‐T buffer. Following this, primary antibodies ([Bax, Bcl‐2, Cyt‐c, Procas‐3 and *β*‐actin]; [1:1000 dilution]) were added and incubated overnight at +4°C. At the end of incubation, membranes were washed three times with TBS‐T for 5 min each and incubated with HRP‐conjugated Affinipure Goat Anti‐Rabbit IgG(H + L) for 90 min (1:2000 dilution) at room temperature. Following the secondary antibody incubation, the membranes underwent three 10‐min TBS‐T washes before the bands were seen using BioRad Clarity Max ECL substrate (Bio‐Rad, CA, USA). Densitometric analysis of the blots was completed using the ImageLab programme (Bio‐Rad, CA, USA).

### 2.6 Statistical Analysis

For statistical analysis, SPSS (Statistical Package for the Social Sciences ver 20.0 SPSS Inc., Chicago, Illinois, USA) was utilized. The results were presented as mean ± standard deviation (SD) after normality analysis. The data was also subjected to a test for homogeneity of variances. One‐way ANOVA and Tukey test as post hoc tests were preferred for comparison between groups. *p* < 0.05, *p* < 0.01 and *p* < 0.001 were considered statistically significant.

## 3 Results

### 3.1 Effects of Vancomycin (Vanco) and Lycopene (Lyco) Treatments on Kidney Function Parameters

**FIGURE 1:**
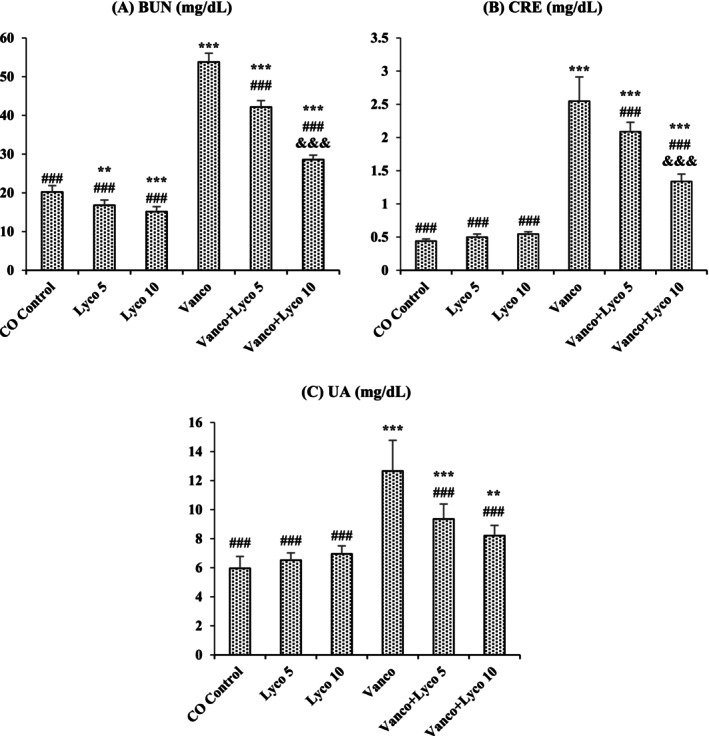
Effects of vancomycin (Vanco) and lycopene (Lyco) administrations on (A) blood urea nitrogen (BUN), (B) creatinine (CRE) and (C) uric acid (UA) levels in serum of rats. Values are given as mean ± SD CO Control versus others: *p < 0.05, **p < 0.01, ***p < 0.001; Vanco versus others: ^#^ *p* < 0.05, ^##^ *p* < 0.01, ^###^ *p* < 0.001; Vanco + Lyco 5 versus Vanco + Lyco 10: ^&^ *p* < 0.05, ^&&^ *p* < 0.01, ^&&&^ *p* < 0.001.

Serum levels of BUN, CRE and UA in Vanco‐treated rats were importantly higher than those in the control group (*p* < 0.001), as shown in Figure 1. In contrast, the increased levels of renal markers in serum were significantly decreased after Lyco treatments at both 5‐ and 10‐mg/kg doses (*p* < 0.001).

### 3.2 Effects of Vanco and Lyco Treatments on Lipid Peroxidation and Antioxidant Defense Parameters

**FIGURE 2:**
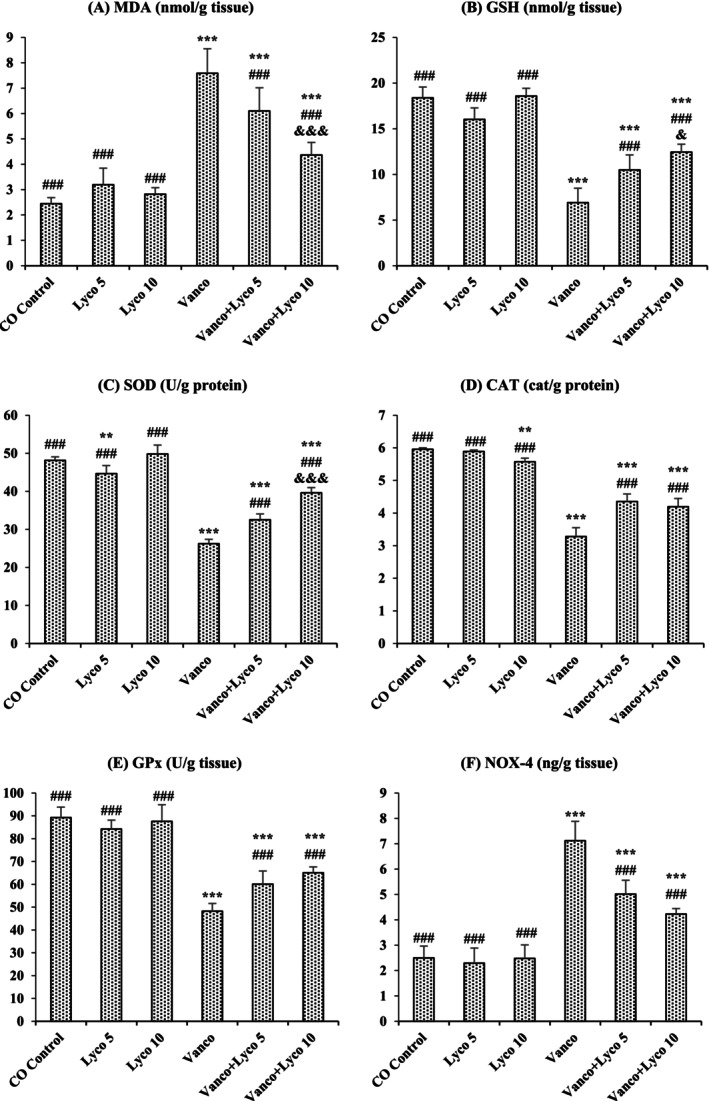
Effects of vancomycin (Vanco) and lycopene (Lyco) administrations on (A) malondialdehyde (MDA), (B) reduced glutathione (GSH), (C) superoxide dismutase (SOD), (D) catalase (CAT), (E) glutathione peroxidase (GPx) and (F) NADPH oxidase 4 (NOX‐4) levels in kidney tissues of rats. Values are given as mean ± SD CO Control versus others: *p < 0.05, **p < 0.01, ***p < 0.001; Vanco versus others: ^#^ *p* < 0.05, ^##^ *p* < 0.01, ^###^ *p* < 0.001; Vanco + Lyco 5 versus Vanco + Lyco 10: ^&^ *p* < 0.05, ^&&^ *p* < 0.01, ^&&&^ *p* < 0.001.

The effects of Vanco and Lyco on MDA, GSH, SOD, CAT, GPx and NOX‐4 parameters in kidney tissue are shown in Figure 2. Compared to the control group, an important decrease in GSH levels and SOD, CAT and GPx activities was ascertained in Vanco‐treated rats (*p* < 0.001). In addition, MDA and NOX‐4 levels in the Vanco group were importantly increased compared to the control group (*p* < 0.001). However, Lyco treatments were found to have a decreasing effect on the elevated MDA and NOX‐4 levels and an increasing effect on the decreased GSH levels due to Vanco administration (*p* < 0.001). In addition, Lyco treatments also increased SOD, CAT and GPx activities (*p* < 0.001).

### 3.3 Effects of Vanco and Lyco Treatments on Inflammatory Parameters

**FIGURE 3:**
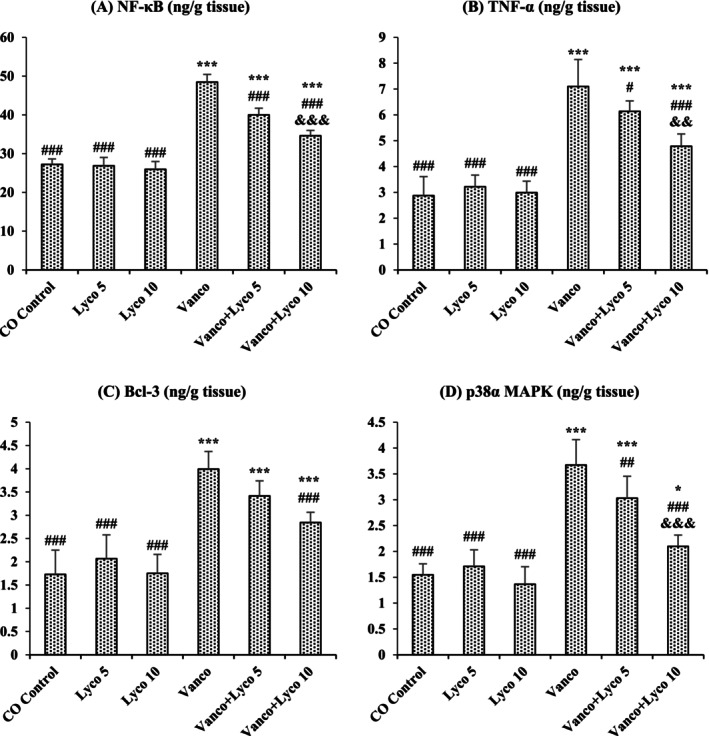
Effects of vancomycin (Vanco) and lycopene (Lyco) administrations on (A) nuclear factor kappa B (NF‐κB), (B) tumour necrosis factor‐alpha (TNF‐*α*), (C) B‐cell lymphoma 3 (Bcl‐3) and (D) mitogen‐activated protein kinase p38 alpha (p38*α* MAPK) levels in kidney tissues of rats. Values are given as mean ± SD CO Control versus others: *p < 0.05, **p < 0.01, ***p < 0.001; Vanco versus others: ^#^ *p* < 0.05, ^##^ *p* < 0.01, ^###^ *p* < 0.001; Vanco + Lyco 5 versus Vanco + Lyco 10: ^&^ *p* < 0.05, ^&&^ *p* < 0.01, ^&&&^ *p* < 0.001.

The effects of Vanco and Lyco on NF‐κB, TNF‐*α*, Bcl‐3 and p38*α* MAPK parameters in kidney tissue are shown in Figure 3. Compared to the control group, NF‐κB, TNF‐*α*, and Bcl‐3 levels and p38*α* MAPK activity were importantly increased in Vanco‐treated rats (*p* < 0.001). The increased levels of these parameters were significantly decreased after Lyco treatments at both 5 and 10 mg/kg doses (*p* < 0.001). There was no critical difference between the control group and the only Lyco‐treated groups.

### 3.4 Effects of Vanco and Lyco Treatments on p53, AQP‐1 and NGAL Levels

**FIGURE 4:**
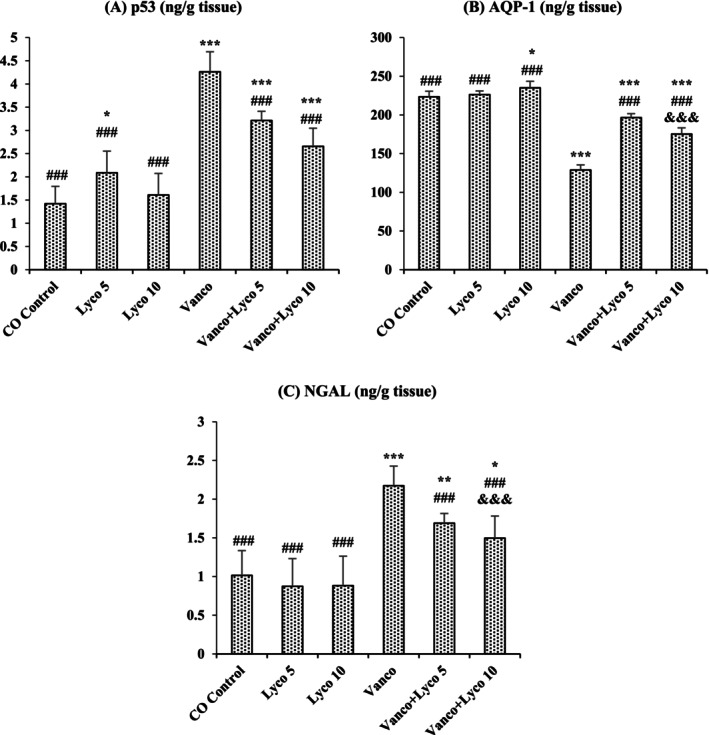
Effects of vancomycin (Vanco) and lycopene (Lyco) administrations on (A) tumour protein 53 (p53), (B) aquaporin 1 (AQP‐1) and (C) neutrophil gelatinase‐associated lipocalin (NGAL) levels in kidney tissues of rats. Values are given as mean ± SD CO Control versus others: *p < 0.05, **p < 0.01, ***p < 0.001; Vanco versus others: ^#^ *p* < 0.05, ^##^ *p* < 0.01, ^###^ *p* < 0.001; Vanco + Lyco 5 versus Vanco + Lyco 10: ^&^ *p* < 0.05, ^&&^ *p* < 0.01, ^&&&^ *p* < 0.001.

The effects of Vanco and Lyco on p53, AQP‐1 and NGAL levels in kidney tissue are shown in Figure 4. Compared to the control group, p53 and NGAL levels were importantly increased, and AQP‐1 levels were importantly decreased in Vanco‐treated rats (*p* < 0.001). However, Lyco treatments showed a decreasing effect on the elevated p53 and NGAL levels and an increasing effect on the decreased AQP‐1 levels due to Vanco administration (*p* < 0.001).

### 3.5 Effect of Vanco and Lyco Treatments on Apoptosis

**FIGURE 5:**
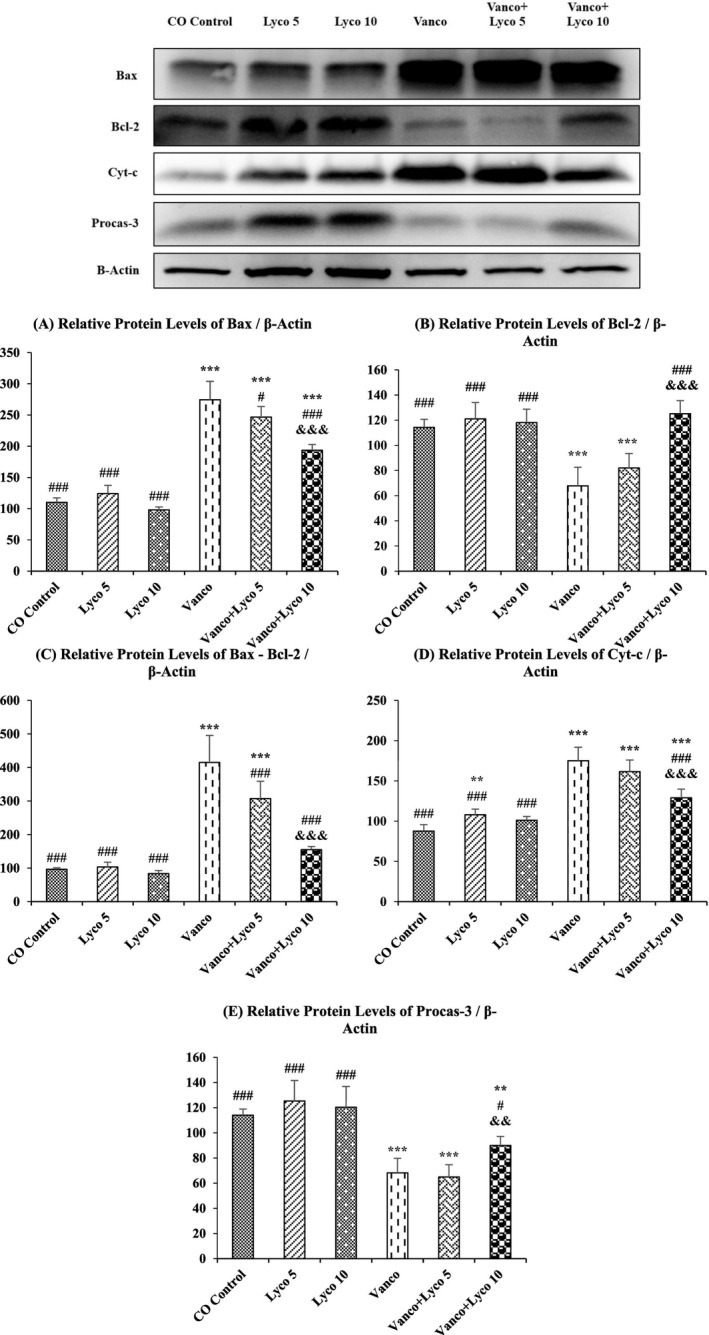
Effects of vancomycin (Vanco) and lycopene (Lyco) administrations on (A) Bcl‐2‐associated X protein (Bax), (B) B‐cell lymphoma 2 (Bcl‐2), (C) ratio of Bax and Bcl‐2, (D) cytochrome c (Cyt‐c) and (E) procaspase‐3 (Procas‐3) levels in kidney tissues of rats. Values are given as mean ± SD (100 fold). CO Control versus others: *p < 0.05, **p < 0.01, ***p < 0.001; Vanco versus others: ^#^ *p* < 0.05, ^##^ *p* < 0.01, ^###^ *p* < 0.001; Vanco + Lyco 5 versus Vanco + Lyco 10: ^&^ *p* < 0.05, ^&&^ *p* < 0.01, ^&&&^ *p* < 0.001.

To further investigate the molecular mechanisms of the anti‐apoptotic effects of Lyco on Vanco‐induced apoptosis in kidney tissue, the expression levels of pro‐apoptotic Bcl‐2‐associated X protein (Bax) and Cytochrome c (Cyt‐c) and anti‐apoptotic B‐cell lymphoma 2 (Bcl‐2) and Procaspase‐3 (Procas‐3) were investigated (Figure 5). We also analysed the Bax/Bcl‐2 ratio, which is critical in determining apoptosis. The Bax/Bcl‐2 ratio was found to be significantly increased with Vanco treatment and decreased with Lyco treatment (*p* < 0.001). Cyt‐c levels increased significantly in the Vanco‐treated group, and an important decrease in Cyt‐c expression level was observed in the Vanco + Lyco 10 group (*p* < 0.001). In addition, Procas‐3 levels were significantly decreased in the Vanco and Vanco + Lyco 5 groups compared to the control group (*p* < 0.001) and were higher in the Vanco + Lyco 10 group compared to these groups (*p* < 0.01).

## 4 Discussion

The kidney is a vital excretion organ of the body. Many substances, such as medicines, as well as internal and external toxins and other toxic metabolites, are eliminated from the body through the kidneys. When some drugs accumulate in the kidney, they can cause kidney damage and kidney failure. Today, the incidence of kidney diseases is increasing. Therefore, drug‐induced NPTs are attracting more and more attention [26].

Vanco binds moderately to plasma proteins, and its distribution volume varies between 0.4 and 1 L/kg. It is excreted in urine without being metabolized. The clearance of the drug is directly proportional to the glomerular filtration rate. The most important side effect of Vanco is NPT [27]. Though the exact mechanism causing Vanco‐induced renal impairment is unknown, recent research indicates that oxidative stress, inflammation, and apoptosis may be linked to the underlying causes of renal damage [7].

In the study, it was determined that BUN, CRE, and UA levels, which are serum biochemical markers of kidney damage [28], showed a significant increase after Vanco administration, and this increase was in parallel with the studies on Vanco administration [2]. The elevated levels of these parameters in the Vanco‐treated group may be a result of the impaired tubular function in the kidney and decreased glomerular filtration rate [29]. The results acquired showed that Lyco (5 and 10 mg/kg) treatment reduced the severity of Vanco‐induced renal damage. Consistent with the data procured, Lyco treatment also improved renal function in the case of arsenic‐induced NPT [30].

There is a balance between reactive oxygen species (ROS) and the elements of the antioxidant defense system (ADS) responsible for ROS elimination. Studies have punctuated that Vanco plays a stimulatory role in oxidative phosphorylation and free radical development by increasing oxygen consumption and cellular ATP concentrations [4]. Various studies have shown a significant increase in MDA level, a lipid peroxidation product, following Vanco administration [31]. In the present study, Vanco significantly increased the MDA level, decreased the activities of SOD, CAT and GPx enzymatic antioxidants, and decreased the GSH level, which is one of the most important non‐enzymatic elements of ADS and shows direct free radical scavenging activity. Moreover, another evidence that Vanco administration induced oxidative damage was the significant increase in NOX‐4 levels. NOX‐4, a NOX isoform, is copiously synthesized in kidney tissue and plays a crucial role in ROS production [32]. Lyco treatment at different doses (5 and 10 mg/kg) decreased Vanco‐induced renal damage by increasing antioxidant enzyme activities and GSH levels and decreasing MDA and NOX‐4 levels. It is stated that carotenoids provide protection against oxidative damage and strengthen the antioxidant system of cells [33]. Lyco is one of the carotenoids with the highest antioxidant properties as it has an open polyene chain without an ionone ring in its molecule [34]. Therefore, the reduction of oxidative stress in kidney tissue due to Vanco administration can be considered normal. Coherent with the data obtained, Lyco treatment reduced the development of oxidative stress in the kidney tissue induced by cisplatin [35] and mercuric chloride [16].

Inflammation is closely associated with oxidative stress resulting from excessive ROS production and inadequate ADS. Inflammatory parameters are thought to play a crucial role in Vanco‐induced NPT [36]. NF‐κB is an important transcription factor known to be sensitive to oxidative stress and is critical in the development of inflammation. It is kept under control through inhibitor kappa B (IκB) [37]. Bcl‐3 is a member of the IκB family and acts as a promoter of transcription factors involved in metabolic pathways for NF‐κB activation [38]. It has been reported that the synthesis of Bcl‐3 increases in kidney injury [39]. IκB phosphorylation can also be activated by p38*α* MAPK. As a result of this activation, the control ability of IκB on NF‐κB is reduced [40]. Liberated NF‐κB regulates the transcription of proinflammatory cytokines such as TNF‐*α* [41]. In the present study, Vanco administration significantly increased NF‐κB, TNF‐*α* and Bcl‐3 levels and p38*α* MAPK activity compared to the control group, while Lyco treatment at two different doses significantly decreased the levels and activity of these inflammatory parameters compared to the Vanco group. Lyco is an antioxidant and anti‐inflammatory carotenoid known for its ability to protect DNA, lipids and proteins from oxidation [42]. The large number of double bonds in its chemical structure is the source of its extraordinary antioxidant activity. Therefore, singlet‐oxygen and peroxyl radicals can be effectively quenched by Lyco [43]. According to the results collected, lipopolysaccharide [44] and 5‐fluorouracil [45] both caused inflammation in kidney tissue, which was lessened by the administration of Lyco therapy.

Aquaporins (AQPs) are transmembrane glycoproteins that allow the entry or release of water through permeable epithelia such as renal tubular epithelium. AQP‐1 is found in the cells of the descending branch of Henle's loop and proximal tubule and contributes to water reabsorption [46]. It has been reported that renal tissue damage affects AQP levels and renal AQP‐1 levels are dramatically reduced in NPT conditions [47]. In this study, it was determined that Vanco had a toxic effect on kidney tissue, causing damage and a decrease in AQP‐1 levels. Then again, it was observed that Lyco treatment significantly increased the AQP‐1 level by decreasing NPT.

In addition to traditional markers of renal damage caused by various factors, NGAL level is considered a more reliable data source for NPT [48]. NGAL, a component of immune responses against infections, is synthesized by renal tubular cells. Damage to these cells causes glomeruli to secrete significant levels of NGAL [49]. In various studies, it has been reported that NGAL levels increase in cases of renal dysfunction [50, 51]. In the study, it was determined that Vanco caused an increase in NGAL levels by damaging the kidney tissue. On the other hand, it was observed that Lyco treatment at both doses significantly decreased the NGAL level compared to the Vanco group by reducing kidney damage.

Apoptosis in nephrocytes is one of the main factors causing renal dysfunction. One of the main causes of Vanco‐induced NPT is apoptotic cell death. It is stated that the main cause of apoptosis due to Vanco administration is increased ROS activity [31]. The p53 protein plays an essential role in the regulation of mitochondria‐dependent apoptotic pathways [52]. In addition, the Bax protein also promotes apoptosis. p53 has been reported to increase Bax synthesis and decrease Bcl‐2 synthesis, which has an anti‐apoptotic character [53]. Depending on the changing Bax/Bcl‐2 ratio, the Cyt‐c level increases in the cytosol. Increased Cyt‐c levels stimulate the apoptotic pathway, accelerating the conversion of Procas‐3 to Caspase‐3. As a result, the synthesis of active caspase‐3 increases and apoptosis is initiated [54]. The western blot analysis performed in this study showed that Vanco caused nephrocyte death by activating p53, Bax, Cyt‐c and Caspase‐3 and decreased Bcl‐2 levels. However, Lyco treatment suppressed p53, Bax and Cyt‐c apoptotic proteins and significantly increased anti‐apoptotic Bcl‐2 and Procas‐3 protein levels. These positive effects were more pronounced in the group treated with Lyco at a dose of 10 mg/kg. Coherent with the data acquired, the positive effects of Lyco administration on apoptosis have been demonstrated by various studies [30, 55].

## 5 Conclusion

This study suggests that Lyco may have the potential to protect kidney tissue from Vanco‐induced damage. The protective activity of Lyco was more effective at a dose of 10 mg/kg. In addition, its capacity to attenuate Vanco‐induced oxidative damage, inflammation, and apoptosis contributed to the search for potential antidotes that could neutralize Vanco‐induced NPT. In conclusion, in addition to Lyco's antioxidant, anti‐inflammatory, and anti‐apoptotic activities, its antidotal activity should also be investigated by further studies.

## Author Contributions

**Hülya Demirkapı Atik:** methodology, data curation, formal analysis, conducted experiments and writing – original draft. **Orkun Atik:** methodology, investigation, formal analysis, conducted experiments and writing and editing. **Recep Aslan:** funding acquisition. **Yavuz Osman Birdane:** conceptualization and supervision. **Abdullah Eryavuz:** supervision.

## Ethics Statement

The National Institutes of Health's ‘Guide for the Care and Use of Laboratory Animals’ was adhered to in all experimental protocols. The Afyon Kocatepe University Animal Experiments Ethics Committee granted approval for the study prior to its implementation (the approval number: 49533702/45).

## Conflicts of Interest

The authors declare no conflicts of interest.

## Data Availability

The data that support the findings of this study are available from the corresponding author upon reasonable request.
